# Territory, neglected diseases and the action of community and endemic combat agents

**DOI:** 10.11606/s1518-8787.2022056003730

**Published:** 2022-04-11

**Authors:** Gabriela Soledad Márdero García, Eliana Amorim de Souza, Vigna Maria de Araújo, Mariana Sousa Santos Macedo, Rosélly Mascarenhas Amaral de Andrade, Paulo Rogers da Silva Ferreira, Maria Cristina Soares Guimarães, José Alexandre Menezes da Silva, Alberto Novaes Ramos

**Affiliations:** I Universidade Federal do Ceará Faculdade de Medicina Programa de Pós-Graduação em Saúde Pública Fortaleza CE Brasil Universidade Federal do Ceará. Faculdade de Medicina. Programa de Pós-Graduação em Saúde Pública. Fortaleza, CE, Brasil; II Universidade Federal da Bahia Instituto Multidisciplinar em Saúde Núcleo Epidemiologia e Saúde Coletiva Vitória da Conquista BA Brasil Universidade Federal da Bahia. Instituto Multidisciplinar em Saúde. Núcleo Epidemiologia e Saúde Coletiva. Vitória da Conquista, BA, Brasil; III Fundação Oswaldo Cruz Instituto de Comunicação e Informação Científica e Tecnológica em Saúde Laboratório de Informação Científica e Tecnológica em Saúde Rio de Janeiro RJ Brasil Fundação Oswaldo Cruz. Instituto de Comunicação e Informação Científica e Tecnológica em Saúde. Laboratório de Informação Científica e Tecnológica em Saúde. Rio de Janeiro, RJ, Brasil; IV Netherlands Hanseniasis Relief do Brasil Fortaleza CE Brasil Netherlands Hanseniasis Relief do Brasil. Fortaleza, CE, Brasil

**Keywords:** Community Health Workers, Neglected Diseases, prevention & control, Leprosy, prevention & control, Health Knowledge, Attitudes, Practice, Primary Health Care

## Abstract

**OBJECTIVE:**

To characterize knowledge, practices, and professional experience of community health agents (ACS) and endemic combat agents (ACE) on leprosy and Chagas disease (DC), during participation in an integrated training workshop in the IntegraDTNs-Bahia project.

**METHODS:**

Descriptive and exploratory case study, involving health agents and endemic combat agents participating in a training workshop on the shared role of these professionals in health care and surveillance processes. The project was developed in the municipalities of Anagé, Tremedal and Vitória da Conquista, in the southwestern State of Bahia, 2019–2020. A specific instrument was applied, with questions related to knowledge and practices of surveillance and care for leprosy and Chagas disease. Descriptive analysis of the data, in addition to consolidation of the lexical analysis, was performed.

**RESULTS:**

Out of a total of 135 participants (107 ACS and 28 ACE), 80.7% of them have been working for at least 12 years, without previous participation in joint training processes. Only 17.9% of endemic combat agentes reported having participated in training on leprosy and none reported developing specific actions to control the disease. For Chagas disease, 36.4% of community health agents participated in training more than a decade before, while for 60.7% of endemic combat agents the last training was carried out in the last five years. The development of educational actions for Chagas disease was more frequent for endemic combat agents (64.3%). When asked about ways of recognizing diseases, the term “skin spots” was the most reported (38 times) for leprosy and, for Chagas disease, the term “I don’t know” (17 times).

**CONCLUSION:**

Processes of health agents and endemic combat agents action in realities endemic for leprosy and Chagas disease in the interior of Bahia proved to be fragmented in the territories. For these diseases, the distance between surveillance and health care actions is reinforced, including in training processes. The importance of innovative permanent and integrated education actions is reiterated to actually promote changes in practices.

## INTRODUCTION

Leprosy and Chagas disease (DC) are infectious and neglected chronic conditions, related to structural poverty as a cause and consequence. Thus, they have a strong potential to cause physical disability, prejudice, stigma and death in territories and populations with greater social vulnerability^1^.

As it presents primary neural involvement by the action of *Mycobacterium leprae*, leprosy is strongly associated with physical disability, which can progress to permanent neuromotor damage to the eyes, hands and feet, if not diagnosed and treated early^2^. According to the World Health Organization (WHO), almost 200 thousand cases of leprosy were registered in 2019, in more than 120 countries, and approximately 15% of cases were registered in Brazil, where about 28 thousand people were diagnosed as new cases^3^.

Similarly, Brazil stands out in the world for the high individual, family and social impact of DC^4^. In its chronic phase, approximately 30–40% of people will develop severe or fatal cardiac, digestive, neurological or mixed changes. In Brazil, the prevalence of infection by *Trypanosoma cruzi* is estimated to range from 1.4 to three million people (0.7–1.4% of the population) in 2020^5^. Chagas disease is one of the four main causes of death from infectious diseases and the Neglected Tropical Disease (NTD) with the highest morbidity in the country^6^. Worldwide, it is estimated that more than seven million people, mainly in Latin America and the Caribbean, are infected with *Trypanosoma cruzi*. Of this total, only 7% were diagnosed and 1% received antiparasitic treatment^5^.

The epidemiological scenario of these two diseases, in addition to the limited availability of diagnostic and therapeutic tools in primary health care (PHC), limited knowledge of the general population and even among health professionals, limited health education initiatives, low social mobilization and stigma, disjointed work processes, among others, brings the challenge of implementing effective and sustainable care actions for people and communities, combined with surveillance actions appropriate to local contexts^6^.

In this scenario, the relevant role of community health agents (ACS) is highlighted, as they are fundamental actors to encourage health promotion in the daily lives of families in their area of activity^9^, as well as the importance of endemic combat agents (ACE) who work in the development of surveillance, prevention and control of diseases related to environmental risk factors^10^. Thus, the need to strengthen health promotion and prevention actions from the apprehension and transformation of territories made the Ministry of Health recommend the insertion of ACE with PHC teams, aiming at shared work^11,12^.

This new logic of work processes organization within the territories attached to health services has required efforts to overcome existing disintegrated models, enabling “new perspectives” of these health professionals and managers at different levels of management^13^. Despite the regulations, in many realities the ACEs have maintained their activities linked to Endemic Control Centers or to the Epidemiological Surveillance structure of the municipalities, coordinated by professionals who do not directly compose the PHC^14^. Therefore, the worrying fragmentation of actions of these professionals in the territories persists, distancing the actions of attention from those of surveillance^15^. One of the critical aspects is the remodeling of continuing education processes for professionals in PHC, focusing on this integration between ACS and ACE, recognizing the specificities but, above all, common aspects in their professional practice.

From this perspective, this work aims to characterize knowledge and practices of ACS and ACE on leprosy and DC, as well as the professional experience of these professionals during participation in a training workshop focused on the integration of surveillance and care actions in PHC in the IntegraDTNs-Bahia Project.

## METHODS

### Study Design

This study is part of the IntegraDTNs-Bahia project, whose objective is to analyze epidemiological and operational patterns on surveillance, prevention and control of leprosy and Chagas disease in municipalities in the Southwest of the State of Bahia, between 2001 and 2018. In this context, the project took on the challenge of identifying knowledge and practices of ACS and ACE on NTDs and, through training processes, sought to present the imperatives of integrating practices in the territory. The project is structured based on a descriptive and exploratory design, based on the experience of ACS and ACE participation in permanent education workshops, inducing the integration of professional practices in the control of DC and leprosy.

The agreed proposal fostered reflections on shared practices, aiming at alternatives for effective changes that would provide spaces for the integration of surveillance, prevention and control actions for the two diseases in the PHC territory.

### Study Location

The study was carried out in the municipalities of Anagé, Tremedal and Vitória da Conquista, located in southwestern Bahia. Vitória da Conquista is the only medium-sized municipality, with an estimated population of 341,128 inhabitants in 2020, constituting itself as a hub city in the health region. Tremedal and Anagé are small municipalities, with a population of 16,189 and 21,607 inhabitants, respectively, in 2020. In all municipalities, PHC is organized based on the Family Health Strategy (FHS), with population coverage ranging from 48.5% (Vitória da Conquista) to 100% (Tremedal and Anagé)^16^. Only Vitória da Conquista has a specialized service for leprosy. For DC, the reference service is located in the city of Salvador, capital of the state. In 2019, the municipalities of Vitória da Conquista, Anagé and Tremedal were characterized as average (8.72 cases/100,000 inhab.), very high (36.96 cases/100,000 inhab.), and high (11.44 cases/100 thousand inhab.) endemicity for leprosy, respectively^17^. Tremedal and Anagé are located in areas of high risk for DC and Vitória da Conquista is in an area of medium risk, due to the history of presence of vectors and reservoirs of *Trypanosoma cruzi* in the region^18^.

### Study Population

Of the total of 661 ACSs and 157 ACEs linked to the Community Health Agents Program (PACS) of the three municipalities, 107 ACSs and 28 ACEs were included, who participated in workshops linked to the IntegraDTNs-Bahia project focusing on the integration between care and surveillance, and who consented to participate in this study.

### Data Collection and Analysis

Data collection and analysis process was integrated with the activities of the workshop for ACS and ACE, with a workload of 16 hours, and conducted between 2019 and 2020. The workshop’s objectives included: 1 - identifying leprosy and DC as public health problems, including aspects related to stigma; 2 - recognize the main clinical characteristics of these diseases, as well as their modes of transmission; 3 - identify the main surveillance and control measures in the PHC territory; and 4 - discuss the integrated role of ACS and ACE in the control of these diseases. The meetings involved an average of 20 participants, always counting on the presence of ACS and ACE.

Within the reception process for the workshop, a collective reading of the Informed Consent Term (ICF) was carried out and, for professionals who agreed to participate, a semi-structured instrument with 39 questions divided into blocks was applied: I) profile of the participants; II) training processes prior to the study and, III) knowledge and practices on surveillance and health care specific to leprosy and DC. This part of the interview precedes the permanent education workshops, that is, it informs about the knowledge before the discussions about leprosy and DC.

Afterwards, the permanent education workshops started, conducted based on active constructivist educational methodologies, including different pedagogical strategies such as conversation circles, case studies, dialogued exhibitions, group work, dramatizations and synthesis.

Participatory strategies were developed seeking to motivate, question, sensitize, mobilize and evaluate the aspects addressed in a participatory way, within the framework of the constructivist theory of Paulo Freire^19^.

To synthesize the reflections made from the triggering question “*How to recognize a person suspected of leprosy and DC?*”, the strategy of individual responses was used, through cards and synthesis words, with the objective of recognizing the reach of information and perceptions about the diseases. Afterwards, there was a moment to measure the recognition of the main characteristics of the clinical syndrome of leprosy and DC, as well as modes of transmission, seeking to build a dialogic learning, through the experiences shared by the agents in their daily realities and within the specificities of their jobs.

The final stage of the workshop was conducted through case studies on the integrated action of these professionals in the development of disease control actions. The method used allowed for a broader analysis based on the stimulus of individual speeches/opinions, followed by discussion and synthesis in small groups and concluding with a plenary session for the discussion of case studies.

Primary data related to the application of the instrument were typed and consolidated, with subsequent descriptive analysis by the Stata software version 11.2, with the construction of tables and graphs. The opinions/considerations of study participants were collected from records written in real time and audio recording (with subsequent transcription), making it possible to systematize each step and the main elements that emerged. For lexicographical analysis, the summaries in documentation (textual corpus) were processed by the free software “INFOGRAM”, available online, and word cloud composition.

### Ethical Considerations

This study was approved by the Research Ethics Committee of the Federal University of Bahia, based on opinion No. 0034/2018. The funding was provided by State of Bahia Research Support Foundation, under Public Notice 003/2017–PPSUS, with additional resources from the *Netherlands Hanseniasis Relief* in Brazil (NHR Brasil), the National Council for Scientific and Technological Development (CNPq), through Universal Call MCTI/CNPq No. 01/2016 by Process No. 433078/2016-2, and the Coordination for the Improvement of Higher Education Personnel (Capes), through the Graduate Support Program (Proap) of the Federal University of Ceará.

## RESULTS

Altogether, 135 professionals participated in the study, including 107 ACS and 28 ACE, most of them female (n = 84; 62.2%), between 41–60 years old (n = 66; 48.9%) and with complete high school (n = 86; 63.7%) (Table). There was a higher frequency of professionals working for at least 12 years in the function (n = 109; 80.7%) and in two work shifts (n = 131; 97.0%). No professionals had participated in training processes in which both professional categories were present. A proportion of 92.5% (n = 99) of ACSs and 17.9% of ACEs (n = 5) reported having participated in training on leprosy, mainly in training courses with a workload of 4–8 hours (n = 66; 48.9%). The development of surveillance and care actions during the home visit was mentioned by 28% (n = 30) of the ACSs and 10.7% (n = 3) of the ACEs. Only ACSs reported developing specific actions for leprosy, with 49.5% (n = 53) reporting having suspected new cases, 47.7% (n = 51) following up on people undergoing treatment, 38.3% (n = 31) searching for contacts of leprosy cases, and 47.7% (n = 51) working on referral of contacts for immunoprophylaxis.

**Table:** Sociodemographic Characteristics of Health Agents who participated in workshops linked to the IntegraDTNs-Bahia Project focusing on the integration between care and surveillance, in municipalities in southwest Bahia, in 2019–2020.

Variables	ACS		ACE		Total	
	n	%	n	%	n	%
Sample	107		28		135	
Municipality						
Anagé	52	48.6	9	32.1	61	45.2
Tremedal	39	36.4	13	46.4	52	38.5
Vitória da Conquista	16	15.0	6	21.4	22	16.3
Sex						
Female	70	65.4	14	50.0	84	62.2
Male	37	34.6	14	50.0	51	37.8
Age						
18–40	49	45.8	15	53.6	64	47.4
41–60	54	50.5	12	42.9	66	48.9
≥ 60	3	2.8	0	0.0	3	2.2
Does not know/does not remember/Did not answer	1	0.9	1	3.6	2	1.5
Length of service						
< 3 years	3	2.8	0	0.0	3	2.2
4–7 years	3	2.8	1	3.6	4	3.0
8–11 years	8	7.5	10	35.7	18	13.3
12–15 years	26	24.3	2	7.1	28	20.7
≥ 16 years	66	61.7	15	53.6	81	60.0
Does not know/does not remember/Did not answer	1	0.9	0	0.0	1	0.7
Scholarship						
4th to 7th grade of elementary school	1	0.9	0	0.0	1	0.7
Complete elementary school	22	20.6	2	7.1	24	17.8
Complete high school	66	61.7	20	71.4	86	63.7
Higher education	18	16.8	6	21.4	24	17.8

ACS: community health agents; ACE: endemic control agents.

**Figure 1:**
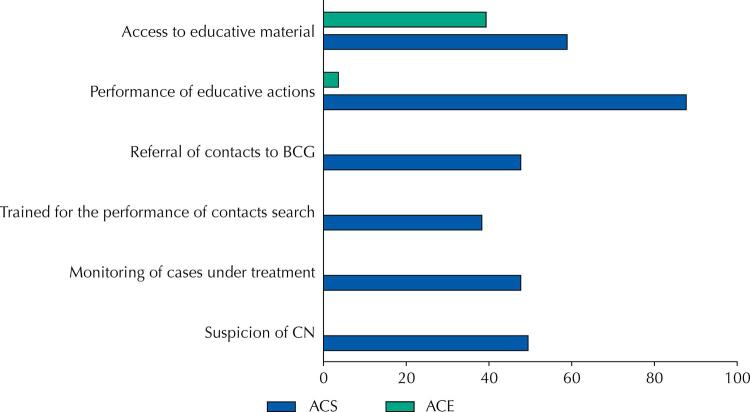
Development of surveillance and health care actions for leprosy in the territory of action of ACS and ACE, in municipalities in southwest Bahia, in 2019–2020.

ACS: community health agents; ACE: endemic control agents; CN: new case.

For most ACS (n = 94; 87.7%) and a small proportion of ACE (n = 1; 3.6%), there was a report of carrying out educational activities on leprosy in the community. Access to specific educational material on the disease was more common among ACSs (n = 63; 58.9%) than among ACEs (n = 11; 39.3%) (Figure 1).

For DC, most ACSs (n = 78; 72.9%) and ACEs (n = 24; 85.7%) reported having already received specific training. However, 36.4% (n = 39) of the ACSs stated that this training took place more than 10 years ago, while for 60.7% of the ACEs (n = 17) the last training was carried out between one and five years. Regardless of the professional category, these trainings lasted a maximum of 8 hours for the majority (n = 80; 59.3%).

Lower proportion of ACS (n = 31; 29%), when compared to ACE (n = 15; 53.6%), frequently carry out home assessments for triatomine capture. Among the ACSs, 6.5% (n = 7) reported having already performed spaying, against 46.4% (n = 13) of the ACEs. The follow-up of people with DC was reported by 10.3% (n = 11) of ACSs, while the referral of suspected cases for diagnostic confirmation was reported by 32.1% (n = 9) of ACEs. These are activities conducted less frequently by ACS (n = 11; 10.3%) than by ACEs (n = 10; 35.7%).

**Figure 2:**
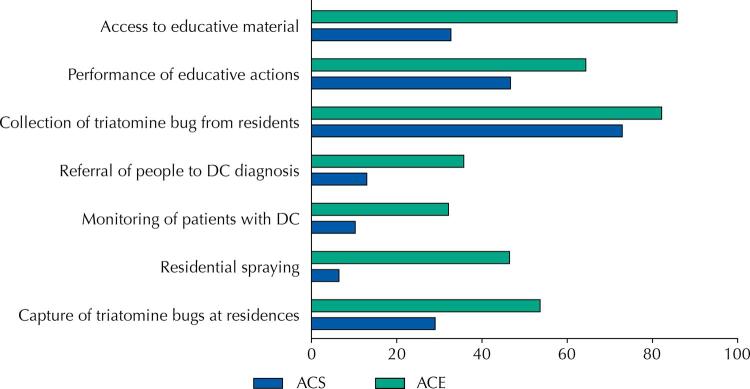
Development of surveillance and health care actions for Chagas disease in the territory of action of ACS and ACE, in municipalities in southwest Bahia, in 2019–2020.

ACS: community health agents; ACE: endemic control agents; DC: Chagas disease.

Passive surveillance activity, characterized by receiving triatomines captured by people in the community, was frequently reported by ACS (n = 78; 72.9%) as well as by ACE (n = 23; 82.1%). On the other hand, the development of educational activities carried out by ACS (n = 50; 46.7%) is lower than for ACEs (n = 18; 64.3%), as well as access to specific educational material, with proportion of 67.3% (n = 72) among ACSs and 85.7% (n = 24) among ACEs, as shown in Figure 2.

During the first moment of the workshop, the reality of the territory in which these professionals work was discussed, presenting some epidemiological and operational indicators, and it was possible to reflect on the magnitude and transcendence of these diseases and possible barriers to the access of care and surveillance actions in the PHC area. Subsequently, during the reports of the professionals’ experiences, it was possible to reflect on the existence of a “stigma” and “imaginary of death” commonly related to leprosy and DC, respectively.

**Figure 3:**
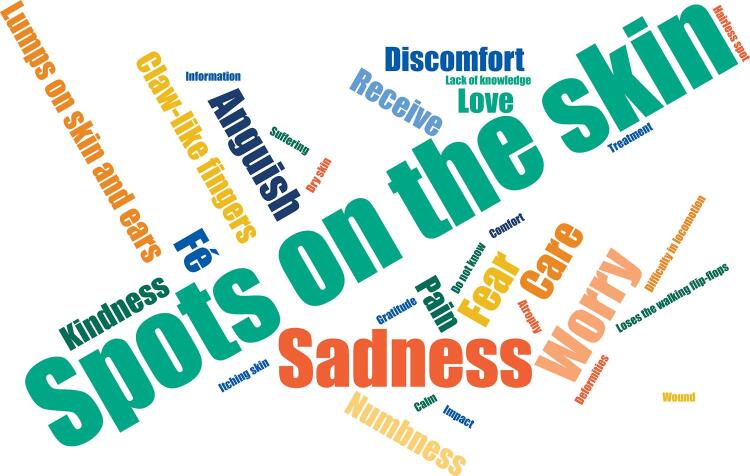
Answers of ACS and ACE to the question “*How to recognize a person suspected of leprosy”*, municipalities in the southwest of Bahia, 2019–2020.

ACS: community health agents; ACE: endemic control agents.

**Figure 4:**
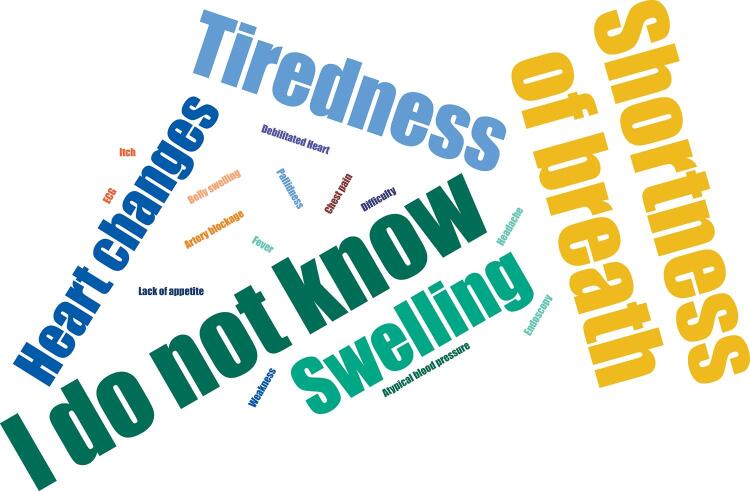
Answers of ACS and ACE to the question “*How to recognize a person suspected of having Chagas disease”*, municipalities in southwest Bahia, 2019–2020.

ACS: community health agents; ACE: endemic control agents.

Figure 3 presents a synthesis of ideas that emerged during this moment of the workshop in response to the question: *How to recognize a person who may have leprosy or DC?* To answer the question, the participants were encouraged to choose a word or term that would answer the question. For leprosy, the term “skin spots” was the most frequently reported (38 times), followed by “loss of sensation” (17 times), “white or reddish spot” (12 times) and “sadness” (nine times). For Chagas disease, the negative “I don’t know” was the most repeated answer (17 times), followed by “shortness of breath” (13 times) and “tiredness” (nine times) (Figure 4).

Using methodological resources, such as flipcharts, videos, triatomine collections, etc., one could recognize correct information about the clinical syndromes of the disease, perspectives of surveillance, prevention, control, and care actions developed exclusively by each of the categories of health professionals, as well as actions that should be and be shared/integrated in order to re-signify perceptions often coated with stigma and to build new knowledge, essential for surveillance and integrated care for these NTDs.

The presentation of ministerial regulations and guidelines on the integration of the work processes of these professionals, particularly the inclusion of ACE in the PHC team, was the subject of debate, allowing the recognition of operational and relational problems that need to be overcome for this integration to occur.

## DISCUSSION

In search of processes that induce integrated surveillance and care practices for leprosy and DC, one could recognize that ACS and ACE, who work in endemic areas for both diseases in the state of Bahia, presented different patterns of knowledge and practice. This finding translates, to a large extent, into guidelines, management and compartmentalized work processes of control and care actions, even among professionals who work in the same territory, with the same people/communities, and with the same diseases. The development process and the experience of participating in health education processes, focusing on the integration between care and surveillance, were significant for rethinking local strategies for coping with these NTDs at the local and national levels^20^.

ACS and ACE are protagonists in the recognition of epidemiological scenarios, including those of transmission of leprosy and DC, as well as other NTDs. The fact these are professionals more inserted in the local territorial context, in the home and community environment, brings great potential for recognizing risk conditions and vulnerabilities for the occurrence of these diseases^13^. The proximity of these professionals to people and their families favors the development of surveillance, care and attention, expanding the possibility of bonding between affected people or people at risk with other members of the PHC team^9^.

One must recognize that health education around leprosy, and especially DC, is still a critical limitation that involves issues such as the identification of the clinical syndrome, diagnostic, treatment, surveillance and control strategies. Thus, the qualification and training process of ACS and ACE has been pointed out as a prominent tool to develop efficient skills to be implemented in the work routine, respecting the singularities of the work process^6,7,13^.

However, this study revealed a clear distinction in the process of developing training actions and practices for the control, promotion and prevention of health for both diseases by ACS and ACE. This also includes, for example, access to educational material for ACSs on leprosy and ACEs on Chagas disease. This revealed that, therefore, regulation in a specific ordinance for the development of integrated actions requires strategies to promote changes in the work process of professionals in the analyzed scenarios. The rupture with rooted models of management and practices requires continuous spaces for reflection, involving the different actors.

Furthermore, the disarticulation between PHC and surveillance and control actions aimed at endemic diseases remain a reality to be faced in these territories^13,21^. Then, it becomes strategic to strengthen the integration of ACE work in activities developed by PHC teams, composing the shared planning of control actions. In fact, the integration of activities developed by ACE and ACS enhances the achievement of goals, qualifying the work process, which becomes even more cooperative^21^.

Faced with these challenges, workshops for the integration of professional practices in the control of DC and leprosy, in this study, were planned as potential spaces to support the critical transformation of ACS and ACE practices. Collective discourses involved in these education processes demonstrated the difficulties of daily life and permeated historical-cultural factors in that location and in our society, including stigma^14,22^. It reaffirms the need for health training that, more than transferring knowledge, should provide the health professional with a practice that is involved with everyday reality^23^.The promotion of open dialogue between ACE and ACS was recognized as an opportunity to experience the real possibility of integrating actions, choosing the territory as a space for action. The power of the meeting pointed to this, but also served to stimulate the emergence of new ideas and possibilities of action, which may or may not complement each other, and which result in the construction of knowledge^24^.

It is worth noting here the revelation of lack of knowledge about specific aspects of the diseases. For DC, “I don’t know” was the most frequent answer, even considering the endemicity of the disease, particularly in Tremedal, one of the residual foci of *Triatoma infestans* in the country^17^. The lack of knowledge of these professionals about the epidemiological relevance of the disease makes it difficult to guarantee access to diagnosis and treatment for affected people, as well as the implementation of more consistent surveillance and control actions for the different modes of transmission.

For leprosy, “spots on the skin” were the most frequently reported, followed by “loss of sensitivity”, “white or reddish spot” and “sadness”. The previous answers corroborate other studies carried out with ACSs on knowledge, attitudes, and practices related to leprosy, in which these professionals also recognize the main clinical signs and symptoms of leprosy, such as skin spots and numbness^25^. It would be important to compare these findings with other studies that specifically included the ACE group and its role in leprosy control; however, the literature is still scarce.

The fact that the fourth mention in the activity was “sadness” makes us reflect on another relevant practice in the care of the subject with leprosy developed by ACS and that should also be developed by ACE: the construction of dialogues with the subjects that included aspects about prejudice and stigma of the disease. Leprosy is still seen as a disease to be feared, due to the physical disability presented by affected and untreated people, generating stigma, disqualification and restriction of social participation, and marginalization^3,4^. Thus, these professionals have a central role in their territories of action, guiding and disseminating accurate information about leprosy, aiming to demystify the negative image associated with it since ancient times^3,17^.

Among the limitations of the study, we point out the limited number of professionals in the municipality of Vitória da Conquista, a result of the stoppage of the workshops due to the pandemic caused by the new coronavirus (covid-19), underway in the country. However, in the case of classically endemic territories, one must also emphasize the necessity to maintain the continuity of spaces for planning and execution of integrated actions between ACE and ACS, enabling the stimulation of the development of these actions as a routine among these professionals during the work, seeking to guarantee the access and quality of the actions developed in the living territory in which social relations are expressed.

## FINAL CONSIDERATIONS

The analysis of knowledge and attitudes reveals the still-existing compartmentalization of surveillance and control actions, translating into a significant distance, including in the training processes. Innovative actions of continuing education in health are increasingly strategic, especially those that focus, from the territory, on the necessary integration of actions and the recognition of overlapping risks and vulnerabilities for different NTDs.

In view of this new sociopolitical context in which the ACEs are inserted and act in a territory shared with the ACS, it becomes central to develop innovative training processes. The proposal developed in this study was guiding and inspiring to rethink local strategies that should be resumed at different times and expanded regionally, initially.

However, we must create spaces, possibilities, and discuss with health managers so that both actors reflect on their practices and discover the attributions that should be shared, respecting the expertise and singularities that mark each of the professions.
