# Supplementary material for: Territory, neglected diseases and the action of community and endemic combat agents
Source: Rev Saude Publica. 2022 Apr 11;56:27. doi: 10.11606/s1518-8787.2022056003730 (PMC9005046; doi:10.11606/s1518-8787.2022056003730)
Supplement: Translation

# Translation: Território, doenças negligenciadas e ação de agentes comunitários e de combate a endemias

**Authors:** Gabriela Soledad Márdero García, Eliana Amorim de Souza, Vigna Maria de Araújo, Mariana Sousa Santos Macedo, Rosélly Mascarenhas Amaral de Andrade, Paulo Rogers da Silva Ferreira, Maria Cristina Soares Guimarães, José Alexandre Menezes da Silva, Alberto Novaes Ramos

**Affiliations:** I Brasil Universidade Federal do Ceará. Faculdade de Medicina. Programa de Pós-Graduação em Saúde Pública. Fortaleza, CE, Brasil; II Brasil Universidade Federal da Bahia. Instituto Multidisciplinar em Saúde. Núcleo Epidemiologia e Saúde Coletiva. Vitória da Conquista, BA, Brasil; III Brasil Fundação Oswaldo Cruz. Instituto de Comunicação e Informação Científica e Tecnológica em Saúde. Laboratório de Informação Científica e Tecnológica em Saúde. Rio de Janeiro, RJ, Brasil; IV Brasil Netherlands Hanseniasis Relief do Brasil. Fortaleza, CE, Brasil

**Keywords:** Agentes Comunitários de Saúde, Doença de Chagas, prevenção & controle, Hanseníase prevenção & controle, Conhecimentos, Atitudes e Prática em Saúde, Atenção Primária à Saúde

## Abstract

**OBJETIVO:**

Caracterizar conhecimentos, práticas e experiência profissional de agentes comunitários de saúde (ACS) e agentes de controle de endemias (ACE) sobre hanseníase e doença de Chagas (DC), durante participação em oficina de formação integrada no projeto IntegraDTNs-Bahia.

**MÉTODOS:**

Estudo de caso descritivo e exploratório, envolvendo comunitários de saúde e agentes de controle de endemias, participantes de oficina de formação sobre o papel compartilhado desses profissionais no processo de vigilância e atenção à saúde. Projeto desenvolvido nos municípios de Anagé, Tremedal e Vitória da Conquista, no Sudoeste do Estado da Bahia, 2019–2020. Aplicou-se instrumento específico prévio com questões relativas a conhecimentos e práticas de vigilância e atenção para hanseníase e doença de Chagas. Análise descritiva dos dados, além de consolidação da análise léxica.

**RESULTADOS:**

Do total de 135 participantes (107 ACS e 28 ACE), 80,7% deles atuam há pelo menos 12 anos, sem participação prévia em processos de formação conjunta. Apenas 17,9% dos agentes de controle de endemias relataram ter participado de capacitações sobre hanseníase e nenhum informou desenvolver ações específicas de controle da doença. Para a doença de Chagas, 36,4% dos agentes comunitários de saúde participaram de capacitações há mais de uma década, enquanto para 60,7% dos agentes de controle de endemias a última capacitação foi realizada nos últimos cinco anos. O desenvolvimento de ações educativas para a doença de Chagas foi mais frequente para agentes de controle de endemias (64,3%). Quando perguntados sobre formas de reconhecimento das doenças, a palavra “manchas na pele” foi a mais relatada (38 vezes) para hanseníase e, para a doença de Chagas, a palavra “não sei” (17 vezes).

**CONCLUSÃO:**

Os processos de atuação de agentes comunitários de saúde e agentes de controle de endemias em realidades endêmicas para hanseníase e doença de Chagas no interior da Bahia revelaram-se desintegrados nos territórios. Para essas doenças, reforça-se o distanciamento entre ações de vigilância e de atenção à saúde, inclusive nos processos de capacitação. Reitera-se a importância de ações inovadoras de educação permanentes e integradas para promover de fato mudanças nas práticas.

## INTRODUÇÃO

A hanseníase e a doença de Chagas (DC) são condições crônicas infecciosas e negligenciadas, relacionadas à pobreza estrutural, como causa e consequência. Assim, apresentam forte potencial para causar incapacidade física, preconceito, estigma e morte em territórios e populações com maior vulnerabilidade social^1^.

Por apresentar acometimento neural primário pela ação de *Mycobacterium leprae*, a hanseníase associa-se fortemente à incapacidade física, que pode evoluir para danos neuro-motores permanentes em olhos, mãos e pés, caso não seja diagnosticada e tratada precocemente^2^. Segundo a Organização Mundial da Saúde (OMS), em 2019 foram registrados quase 200 mil casos de hanseníase em mais de 120 países, aproximadamente 15% dos casos estavam registrados no Brasil, cerca de 28 mil pessoas diagnosticadas como caso novo^3^.

De igual forma, o Brasil destaca-se no mundo pelo elevado impacto individual, familiar e social da DC^4^. Em sua fase crônica, aproximadamente 30–40% das pessoas desenvolverão alterações cardíacas, digestivas, neurológicas ou mistas, graves ou fatais. Estima-se no Brasil uma prevalência de infecção por *Trypanosoma cruzi*, em 2020, de 1,4 a três milhões de pessoas (0,7–1,4% da população)^5^. É uma das quatro principais causas de morte por doenças infecciosas e a Doença Tropical Negligenciada (DTN) com maior morbidade no país^6^. Em todo o mundo, estima-se que mais de sete milhões de pessoas, principalmente na América Latina e Caribe, estejam infectadas por *Trypanosoma cruzi*. Desse total, presume-se que apenas 7% foram diagnosticadas e 1% recebeu tratamento antiparasitário^5^.

O cenário epidemiológico dessas duas doenças, além da limitada disponibilidade de ferramentas diagnósticas e terapêuticas na atenção primaria à saúde (APS), conhecimento restrito da população geral e mesmo entre profissionais de saúde, iniciativas limitadas de educação em saúde, baixa mobilização social e estigma, processos desarticulados de trabalho, entre outras, traz o desafio de implementar ações efetivas e sustentáveis de cuidado às pessoas e às comunidades, aliadas às ações de vigilância adequadas aos contextos locais^6^.

Nesse cenário, ressalta-se o relevante papel de agentes comunitários de saúde (ACS), atores fundamentais para o incentivo à promoção da saúde no cotidiano das famílias de sua área de atuação^9^, assim como é reconhecida a importância dos agentes de combate a endemias (ACE) que atuam no desenvolvimento de ações de vigilância, prevenção e controle de doenças relacionadas a fatores ambientais de risco^10^ Assim, a necessidade de fortalecimento das ações de promoção e prevenção da saúde a partir da apreensão e transformação dos territórios, fez com que o Ministério da Saúde recomendasse a inserção de ACE junto às equipes da APS, visando trabalho compartilhado^11,12^.

Essa nova lógica de organização dos processos de trabalho, dentro dos territórios adstritos dos serviços de saúde, tem exigido esforços para superar modelos desintegrados vigentes, possibilitando “novos olhares” desses profissionais de saúde e de gestores dos diferentes níveis de gestão^13^. Apesar das normativas, em muitas realidades os ACE têm mantido atuação vinculada a Centros de Controle de Endemias ou à estrutura de Vigilância Epidemiológica dos municípios, coordenados por profissionais que não compõem diretamente a APS^14^. Persiste, portanto, a preocupante fragmentação das ações desses profissionais nos territórios, distanciando as ações de atenção das de vigilância^15^. Um dos aspectos críticos é o remodelamento dos processos de educação permanente do profissional na APS, focando essa integração entre ACS e ACE, reconhecendo-se as especificidades, mas, sobretudo, os aspectos comuns em sua prática profissional.

Nessa perspectiva, este trabalho tem como objetivo caracterizar conhecimentos e práticas de ACS e ACE, sobre hanseníase e DC, e a experiência profissional desses profissionais durante participação em oficina de formação com foco na integração de ações de vigilância e atenção na APS no projeto IntegraDTNs-Bahia.

## MÉTODOS

### Desenho de Estudo

O presente estudo compõe o projeto IntegraDTNs-Bahia, cujo objetivo é analisar padrões epidemiológicos e operacionais sobre vigilância, prevenção e controle da hanseníase e da doença de Chagas em municípios do Sudoeste do Estado da Bahia, entre 2001 e 2018. Nesse contexto, o projeto assumiu o desafio de identificar conhecimentos e práticas de ACS e ACE sobre DTN e, por meio de processos formativos, procurou apresentar os imperativos da integração das práticas no território. Foi estruturado com base em um desenho descritivo e exploratório, fundamentado na experiência de participação de ACS e ACE em oficinas de educação permanente, indutoras da integração das práticas profissionais no controle da DC e da hanseníase.

A proposta pactuada fomentou reflexões de práticas compartilhadas, visando alternativas para mudanças efetivas que oportunizassem espaços para integração de ações de vigilância, prevenção e controle das duas doenças no território da APS.

### Local do Estudo

O estudo foi desenvolvido nos municípios de Anagé, Tremedal e Vitória da Conquista, localizados no Sudoeste do Estado da Bahia. Vitória da Conquista é o único município de porte médio, com população estimada em 2020 de 341.128 habitantes, constituindo-se como cidade polo da região de saúde. Tremedal e Anagé são municípios de pequeno porte, com população de 16.189 e 21.607 habitantes, respectivamente, em 2020. Em todos os municípios, a APS está organizada a partir da Estratégia Saúde da Família (ESF), com coberturas populacionais variando de 48,5% (Vitória da Conquista) a 100% (Tremedal e Anagé)^16^. Apenas Vitória da Conquista dispõe de serviço especializado para hanseníase. Para a DC, o serviço de referência localiza-se no município de Salvador, capital do estado. Em 2019, os municípios de Vitória da Conquista, Anagé e Tremedal se caracterizaram em média (8,72 casos/100 mil hab.), muito alta (36,96 casos/100 mil hab.), e alta (11,44 casos/100 mil hab.) endemicidade para hanseníase respectivamente^17^. Tremedal e Anagé, estão localizadas em áreas de elevado risco para DC e Vitória da Conquista, de médio risco, devido ao histórico da presença de vetores e reservatórios de *Trypanosoma cruzi* na região^18^.

### População do Estudo

Do total de 661 ACS e 157 ACE vinculados ao Programa de Agentes Comunitários de Saúde (PACS) dos três municípios, foram incluídos 107 ACS e 28 ACE que participaram de oficinas vinculadas ao projeto IntegraDTNs-Bahia, com foco na integração entre atenção e vigilância, e que consentiram em participar deste estudo.

### Coleta e Análise de Dados

O processo de coleta e análise de dados foi integrado às atividades da oficina de trabalho para ACS e ACE, com carga horária de 16 horas, e conduzidas nos anos de 2019 e 2020. Entre os objetivos da oficina, inseriam-se: 1 - identificar a hanseníase e a DC como problemas de saúde pública, incluindo aspectos relacionados ao estigma; 2 - reconhecer as principais características clínicas dessas doenças, assim como os seus modos de transmissão; 3 - identificar as principais medidas de vigilância e controle no território da APS; e 4 - discutir o papel integrado dos ACS e ACE no controle dessas doenças. Os encontros envolveram em média 20 participantes, contando sempre com a presença de ACS e ACE.

Dentro do processo de acolhimento para a oficina, foi realizada a leitura coletiva do Termo de Consentimento Livre Esclarecido (TCLE) e, para os profissionais que aceitaram participar, aplicou-se instrumento semiestruturado com 39 perguntas divididas em blocos: I) perfil dos participantes; II) processos de capacitação anteriores ao estudo e, III) conhecimentos e práticas sobre vigilância e atenção à saúde específicos para hanseníase e DC. Essa parte da entrevista é anterior à oficina de educação permanente, ou seja, informa sobre o conhecimento antes das discussões sobre hanseníase e DC.

Na sequência, seguiu-se para a oficina de educação permanente, conduzida com base em metodologias educacionais construtivistas ativas, incluindo diferentes estratégias pedagógicas, como rodas de conversa, estudos de caso, exposições dialogadas, trabalhos em grupo, dramatizações e síntese.

Foram desenvolvidas estratégias participativas buscando motivar, questionar, sensibilizar, mobilizar e avaliar de modo participativo os aspectos abordados, dentro dos referenciais da teoria construtivista de Paulo Freire^19^.

Para síntese das reflexões realizadas a partir da questão disparadora “*Como reconhecer uma pessoa suspeita de hanseníase e de DC?*”, utilizou-se a estratégia de respostas individuais, por meio de tarjetas e palavras-síntese, tendo como objetivo reconhecer o alcance de informações prévias e percepções sobre as doenças. Depois, seguiu-se um momento para medir o reconhecimento das principais características da síndrome clínica da hanseníase e da DC, assim como modos de transmissão, buscando construir uma aprendizagem dialógica, mediante as vivências compartilhadas pelos agentes em suas realidades cotidianas e dentro das especificidades de seus trabalhos.

A etapa final da oficina foi conduzida por estudos de casos sobre atuação integrada desses profissionais no desenvolvimento de ações de controle das doenças. O método utilizado permitiu uma análise mais ampla a partir do estímulo de falas/opiniões individuais, seguido de discussão e síntese em pequenos grupos e concluindo com plenária para discussão sobre os estudos de caso.

Os dados primários relativos à aplicação do instrumento foram digitados e consolidados, com posterior análise descritiva pelo software Stata versão 11.2, com a construção de tabelas e gráficos. As opiniões/considerações de participantes do estudo foram coletadas a partir do registro escrito em tempo real e gravação de áudio (com posterior transcrição), possibilitando sistematizar cada etapa e os principais elementos que emergiram. Para análise lexicográfica, as sínteses em documentação (corpus textuais) foram processadas pelo software gratuito disponível on-line “INFOGRAM” e composição de nuvem de palavras.

### Considerações Éticas

O presente estudo foi aprovado pelo Comitê de Ética em Pesquisa da Universidade Federal da Bahia, com base no parecer n^o^ 0034/2018. Financiamento pela Fundação de Amparo à pesquisa do Estado da Bahia, Edital 003/2017–PPSUS, com recursos adicionais oriundos da *Netherlands Hanseniasis Relief* do Brasil (NHR Brasil), do Conselho Nacional de Desenvolvimento Científico e Tecnológico (CNPq), via Chamada Universal MCTI/CNPq nº 01/2016 pelo Processo nº 433078/2016-2 e da Coordenação de Aperfeiçoamento de Pessoal de Nível Superior (Capes), via Programa de Apoio à Pós-Graduação (Proap) da Universidade Federal do Ceará.

## RESULTADOS

Ao todo, participaram do estudo 135 profissionais, dentre eles 107 ACS e 28 ACE, a maioria do sexo feminino (n = 84; 62,2%), idade entre 41–60 anos (n = 66; 48,9%) e com ensino médio completo (n = 86; 63,7%) (Tabela). Houve maior frequência de profissionais que atuam há pelo menos 12 anos na função (n = 109; 80,7%) e em dois turnos de trabalho (n = 131; 97,0%). Nenhum profissional havia participado de processos de formação em que havia presença das duas categorias profissionais. Uma proporção de 92,5% (n = 99) dos ACS e 17,9% dos ACE (n = 5) relataram ter participado de capacitações sobre hanseníase, principalmente em formações com carga horária de 4–8 horas (n = 66; 48,9%). O desenvolvimento de ações de vigilância e cuidado durante a visita domiciliar foi mencionado por 28% (n = 30) dos ACS e 10,7% (n = 3) dos ACE. Somente os ACS relataram desenvolver ações específicas para hanseníase, sendo que 49,5% (n = 53) declararam realizar suspeição de casos novos, 47,7% (n = 51) acompanhamento de pessoas em tratamento, 38,3% (n = 31), busca de contatos de casos de hanseníase e 47,7% (n = 51) encaminhamento dos contatos para imunoprofilaxia.

**Tabela:** Características sociodemográficas dos agentes de saúde que participaram de oficinas vinculadas ao projeto IntegraDTNs-Bahia com foco na integração entre atenção e vigilância,em municípios do sudoeste da Bahia, em 2019–2020.

Variáveis	ACS		ACE		Total	
		
	n	%	n	%	n	%
Amostra	**107**		**28**		**135**	
Município						
Anagé	52	48,6	9	32,1	61	45,2
Tremedal	39	36,4	13	46,4	52	38,5
Vitória da Conquista	16	15,0	6	21,4	22	16,3
Sexo						
Feminino	70	65,4	14	50,0	84	62,2
Masculino	37	34,6	14	50,0	51	37,8
Idade						
18–40	49	45,8	15	53,6	64	47,4
41–60	54	50,5	12	42,9	66	48,9
≥ 60	3	2,8	0	0,0	3	2,2
Não lembra/não sabe/não quer responder	1	0,9	1	3,6	2	1,5
Tempo de atuação						
< 3 anos	3	2,8	0	0,0	3	2,2
4–7 anos	3	2,8	1	3,6	4	3,0
8–11 anos	8	7,5	10	35,7	18	13,3
12–15 anos	26	24,3	2	7,1	28	20,7
≥ 16 anos	66	61,7	15	53,6	81	60,0
Não lembra/não sabe/não quer responder	1	0,9	0	0,0	1	0,7
Escolaridade						
4ª a 7ª série do ensino fundamental	1	0,9	0	0,0	1	0,7
Ensino fundamental completo	22	20,6	2	7,1	24	17,8
Ensino médio completo	66	61,7	20	71,4	86	63,7
Ensino superior	18	16,8	6	21,4	24	17,8

ACS: agentes comunitários de saúde; ACE: agentes de controle de endemias.

Para a maioria de ACS (n = 94; 87,7%) e uma pequena proporção de ACE (n = 1; 3,6%) houve relato de realização de ações educativas sobre hanseníase junto à comunidade. O acesso a material educativo específico sobre a doença foi mais comum entre ACS (n = 63; 58,9%) do que entre ACE (n = 11; 39,3%) (Figura 1).

**Figura 1:** Desenvolvimento de ações de vigilância e cuidado à saúde para hanseníase no território de atuação de ACS e ACE, em municípios do sudoeste da Bahia, em 2019–2020.

ACS: agentes comunitários de saúde; ACE: agentes de controle de endemias; CN: caso novo.

Para a DC, a maioria dos ACS (n = 78; 72,9%) e dos ACE (n = 24; 85,7%) relatou já ter recebido capacitação específica. No entanto, 36,4% (n = 39) dos ACS afirmaram que essa formação ocorreu há mais de 10 anos, enquanto para 60,7% dos ACE (n = 17) a última capacitação foi realizada entre um e cinco anos. Independentemente da categoria profissional, essas formações tiveram duração máxima de 8 horas para a maioria (n = 80; 59,3%).

Proporção menor de ACS (n = 31; 29%), quando comparados a ACE (n = 15; 53,6%), realiza frequentemente avaliação domiciliar para captura de triatomíneos. Entre os ACS, 6,5% (n = 7) informou já ter realizado borrifação, contra 46,4% (n = 13) dos ACE. O acompanhamento de pessoas com DC foi referido por 10,3% (n = 11) desses profissionais, enquanto o encaminhamento de casos suspeitos para confirmação diagnóstica foi referido por 32,1% (n = 9). Trata-se de atividades conduzidas menos frequentemente por ACS (n = 11; 10,3%) do que por ACE (n = 10; 35,7%).

A atividade de vigilância passiva, caracterizada por recebimento de triatomíneos capturados por pessoas da comunidade, foi frequentemente relatada por ACS (n = 78; 72,9%) como também por ACE (n = 23; 82,1%). Por outro lado, o desenvolvimento de ações educativas realizada por ACS (n = 50; 46,7%) é menor do que para os ACE (n = 18; 64,3%), assim como o acesso a material educativo específico, com proporção de 67,3% (n = 72) entre ACS e de 85,7% (n = 24) entre ACE, conforme apresentado na Figura 2.

**Figura 2:** Desenvolvimento de ações de vigilância e cuidado à saúde para doença de Chagas no território de atuação de ACS e ACE, em municípios do sudoeste da Bahia, em 2019–2020.

ACS: agentes comunitários de saúde; ACE: agentes de controle de endemias; DC: doença de Chagas.

Durante o primeiro momento da oficina discutiu-se a realidade do território de atuação desses profissionais, apresentando alguns indicadores epidemiológicos e operacionais foi possível refletir sobre a magnitude e transcendência dessas doenças e possíveis barreiras de acesso às ações de cuidado e vigilância na área da APS. Na sequência, durante os relatos de vivências dos profissionais, foi possível refletir sobre a existência do “estigma” e do “imaginário de morte” comumente relacionados à hanseníase e à DC, respectivamente.

A Figura 3 apresenta uma síntese de ideias que emergiram durante esse momento da oficina como resposta à questão: *Como reconhecer uma pessoa que possa ter hanseníase ou DC?* Para responderem à pergunta, os participantes foram instigados a escolher uma palavra ou termo que respondesse à indagação. Para hanseníase, o termo “manchas na pele” foi o mais frequentemente relatado (38 vezes), seguido de “perda da sensibilidade” (17 vezes), “mancha esbranquiçada ou avermelhada” (12 vezes) e “tristeza” (nove vezes). Para a doença de Chagas a negativa “não sei” foi a resposta mais repetida (17 vezes), seguida por “falta de ar” (13 vezes) e “cansaço” (nove vezes) (Figura 4).

**Figura 3:** Resposta direcionada por ACS e ACE à questão “*Como reconhecer uma pessoa suspeita de hanseníase*”, municípios do sudoeste da Bahia, 2019–2020.

ACS: agentes comunitários de saúde; ACE: agentes de controle de endemias.

**Figura 4:** Resposta direcionada por ACS e ACE à questão “*Como reconhecer uma pessoa suspeita de doença de Chagas*”, municípios do sudoeste da Bahia, 2019–2020.

ACS: agentes comunitários de saúde; ACE: agentes de controle de endemias.

Com uso de recursos metodológicos, como álbum seriado, vídeos, coleção de triatomíneos etc., foi possível reconhecer informações corretas sobre as síndromes clínicas da doença, perspectivas das ações de vigilância, prevenção, controle e cuidado desenvolvidas exclusivamente por cada uma das categorias de profissionais de saúde, assim como ações que deveriam ser e estar compartilhadas/integradas visando ressignificar percepções muitas vezes revestidas por estigma e construir novos conhecimentos, essenciais para a vigilância e cuidado integrado a essas DTN.

A apresentação de normativas e diretrizes ministeriais sobre a integração dos processos de trabalho desses profissionais, particularmente a inclusão de ACE na equipe da APS, foi tema de debate, permitindo reconhecer problemas operacionais e relacionais que precisam ser superados para que ocorra essa integração.

## DISCUSSÃO

Em busca de processos indutores de práticas integradas de vigilância e cuidado para a hanseníase e DC, foi possível reconhecer que ACS e ACE, que atuam em áreas endêmicas para ambas as doenças no estado da Bahia, apresentaram padrões diferenciados de conhecimento e de prática. Essa constatação traduz, em grande parte, diretrizes, gestão e processos de trabalho compartimentalizados das ações de controle e cuidado, inclusive entre profissionais que atuam no mesmo território, com as mesmas pessoas/comunidades e com as mesmas doenças. O processo de desenvolvimento e a experiência de participação em processos de educação em saúde, com foco na integração entre atenção e vigilância, foram significativos para se repensar estratégias locais de enfrentamento dessas DTN no plano local e nacional^20^.

Agentes comunitários de saúde e agentes de controle de endemias são protagonistas no reconhecimento de cenários epidemiológicos, incluindo os de transmissão da hanseníase e DC, assim como de outras DTN. O fato de serem profissionais mais inseridos no contexto territorial local, no ambiente domiciliar e comunitário, traz um grande potencial para reconhecimento de condições de risco e vulnerabilidades para ocorrência dessas doenças^13^. A proximidade desses profissionais com as pessoas e suas famílias favorece o desenvolvimento de ações de vigilância, cuidado e atenção, ampliando a possibilidade de vínculo entre as pessoas acometidas ou sob risco com os demais membros da equipe da APS^9^.

Reconhece-se que a educação em saúde em torno da hanseníase e principalmente da DC, ainda é uma limitação crítica que envolve questões como a identificação da síndrome clínica, bem como das estratégias de diagnóstico, tratamento, vigilância e controle. Assim, o processo de capacitação e treinamento de ACS e ACE tem sido apontado como ferramenta de destaque para desenvolver habilidades eficientes a serem implementadas na rotina laboral, respeitando as singularidades do processo de trabalho^6,7,13^

Entretanto, o presente estudo revelou uma clara distinção no processo de desenvolvimento das ações de formação e de práticas para o controle, promoção e prevenção à saúde para as duas doenças por parte de ACS e ACE. Isso inclui também, por exemplo, o acesso a material educativo dos ACS para a hanseníase e dos ACE para a DC. Revela-se, portanto, que a normatização em portaria específica para desenvolvimento de ações integradas, exige estratégias para promover mudanças no processo de trabalho dos profissionais nos cenários analisados. A ruptura com modelos enraizados de gestão e de práticas exige espaços contínuos de reflexão, envolvendo os diferentes atores.

Ademais, a desarticulação entre a APS e as ações de vigilância e controle direcionadas para endemias permanecem como realidade a ser enfrentada nesses territórios^13,21^. Torna-se, então, estratégico o fortalecimento da integração do trabalho de ACE em atividades desenvolvidas pelas equipes da APS, compondo o planejamento compartilhado de ações de controle. De fato, a integração das atividades desenvolvidas por ACE e ACS potencializa o alcance de metas, qualificando o processo de trabalho que passa a se tornar ainda mais cooperativo^21^.

Diante desses desafios, as oficinas de trabalho para integração das práticas profissionais no controle da DC e da hanseníase deste estudo foram planejadas como espaços potenciais para apoio à transformação crítica das práticas de ACS e ACE. Os discursos coletivos envolvidos nesses processos de educação demonstraram as dificuldades do cotidiano e permearam fatores histórico-culturais daquela localidade e de nossa sociedade, inclusive do estigma^14,22^. Reafirma-se, a necessidade de uma formação em saúde que, mais do que transferir conhecimentos, deve proporcionar ao profissional da saúde uma prática implicada com a realidade cotidiana^23^.Reconheceu-se a promoção do diálogo aberto entre ACE e ACS como uma oportunidade de experimentar a real possibilidade de integrar as ações, elegendo o território como espaço de atuação. A potência do encontro apontou para isso, mas também para estímulo ao surgimento de novas ideias e possibilidades de atuação, que podem ou não se complementar, e que resultam na construção de novos conhecimentos^24^.

Ressalta-se aqui a revelação de desconhecimento sobre aspectos próprios das doenças. Para a DC, “não sei” foi a resposta mais frequentemente, mesmo considerando-se o caráter de endemicidade para a doença, particularmente Tremedal, um dos focos residuais de *Triatoma infestans* no país^17^. O desconhecimento desses profissionais sobre a relevância epidemiológica da doença dificulta a garantia do acesso a diagnóstico e tratamento das pessoas acometidas, bem como a efetivação de ações de vigilância e controle mais consistentes, para os diferentes modos de transmissão.

Para hanseníase, “manchas na pele” foi a mais frequentemente relatada, seguida de “perda da sensibilidade”, “mancha esbranquiçada ou avermelhada” e “tristeza”. As respostas anteriores corroboram outros estudos realizados com ACS sobre conhecimentos, atitudes e práticas relacionados à hanseníase, em que esses profissionais reconhecem também os principais sinais e sintomas clínicos da hanseníase, como manchas na pele e dormência^25^. Seria importante comparar estes achados com outros estudos que tivessem incluído especificamente o grupo de ACE e sua atuação no controle da hanseníase, porém, a literatura ainda é escassa.

O fato de a quarta menção na atividade ter sido “tristeza” nos faz refletir sobre outra prática relevante no cuidado ao sujeito com hanseníase desenvolvida por ACS e que também deveria ser desenvolvida por ACE: a construção de diálogos com os sujeitos que incluíssem aspectos sobre preconceito e estigma da doença. A hanseníase ainda é vista como uma doença a ser temida, devido à incapacidade física apresentada pelas pessoas acometidas e não tratadas, gerando estigma, desqualificação e restrição à participação social e marginalização^3,4^. Assim, esses profissionais têm papel central nos territórios de atuação, de orientar e divulgar informações precisas sobre a hanseníase, visando desmistificar a imagem negativa associada desde tempos remotos^3,17,^.

Entre as limitações do estudo, apontamos o número limitado de profissionais do município de Vitória da Conquista, resultado da paralisação das oficinas devido à pandemia da doença causada pelo novo coronavírus (covid-19) em curso no país. Contudo, tratando-se de territórios classicamente endêmicos, enfatiza-se, ainda, que é necessário manter a continuidade dos espaços de planejamento e execução de ações integradas entre ACE e ACS, possibilitando o estímulo ao desenvolvimento dessas ações como rotina entre esses profissionais durante o trabalho, buscando garantir o acesso e a qualidade das ações desenvolvidas no território vivo em que se expressam as relações sociais.

## CONSIDERAÇÕES FINAIS

A análise de conhecimento e atitudes revela a compartimentalização ainda vigente das ações de vigilância e controle traduzindo-se em significativo distanciamento, inclusive nos processos de capacitação. Ações inovadoras de educação permanente em saúde são cada vez mais estratégicas, principalmente as que enfoquem, a partir do território, a necessária integração de ações e o reconhecimento da sobreposição de riscos e vulnerabilidades para diferentes DTN.

Diante desse novo contexto sociopolítico em que os ACE se inserem e atuam em um território compartilhado com os ACS, torna-se central desenvolver processos de formação inovadores. A proposta desenvolvida neste estudo foi orientadora e inspiradora para se repensar estratégias locais que devem ser retomadas em distintos momentos e ampliadas regionalmente, em uma primeira perspectiva.

No entanto, é preciso criar espaços, possibilidades e discutir com gestores em saúde para que os dois atores reflitam sobre suas práticas e descubram as atribuições que devam ser compartilhadas, respeitando as expertises e singularidades que marcam cada uma das profissões.
